# High‐Performance Micro‐LED Displays via Etching‐Damage‐Free Pixelation Strategy for Multifunctional Integrated Applications

**DOI:** 10.1002/advs.202511520

**Published:** 2025-09-03

**Authors:** Jinyu Ye, Wenjuan Su, Yibin Lin, Yuyan Peng, Xiongtu Zhou, Tailiang Guo, Jiade Yuan, Jie Sun, Qun Yan, Yongai Zhang, Chaoxing Wu

**Affiliations:** ^1^ College of Physics and Information Engineering of Fuzhou University Fuzhou Fujian 350108 P. R. China; ^2^ Fujian Science & Technology Innovation Laboratory for Optoelectronic Information of China Fuzhou Fujian 350108 P. R. China

**Keywords:** Etching‐damage‐free pixelation strategy, Ion implantation, Micro‐LED display, Multifunctional integrated applications

## Abstract

As Micro‐LED sizes shrink, luminescence efficiency drops significantly due to sidewall damage from plasma etching. This study introduces a precision‐selective ion implantation (PSII) strategy to boost external quantum efficiency (EQE) and brightness of Micro‐LED at high current density, vital for applications like augmented reality (AR) and optical communication, instead of relying on sidewall passivation for low current density efficiency. PSII's effects is systematically evaluated on electrical isolation and photoelectric properties. Results demonstrate that Micro/Nano‐LED arrays with a pixel density up to 25,400 ppi are achieved through the PSII strategy. Turn‐off leakage current is reduced, with improved carrier injection efficiency, EQE, and luminance. At 10,000 A cm^−^^2^, EQE and brightness increased by ≈30% and ≈25%, respectively. Moreover, lattice damage induced by ion bombardment enhanced UV and blue light absorption while suppressing visible emission, which benefits photodetectors (PDs). Demonstrations in AR displays, optical communication systems, and UV PDs showed superior performance compared to etching‐based methods. The modulation bandwidth reached 131.2 MHz, a 30% improvement, and PD photocurrent increased by ≈ 90%. This PSII‐based pixelation strategy enables high‐brightness Micro‐LED displays with significant potential for multifunctional integrated applications, offering a robust alternative to traditional etching processes for advanced optoelectronic devices.

## Graphical abstract

A novel etching‐damage‐free pixelation approach based on F ion implantation has been developed for the fabrication of micro/nano‐LED arrays with a pixel density reaching 25,400 ppi. This method offers the advantages of high resolution, high efficiency, and high brightness, making it highly suitable for applications in near‐eye displays, optical communications, and photodetectors, and holding great promise for multifunctional integrated systems.

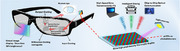

## 1 Introduction

**Figure 1:**
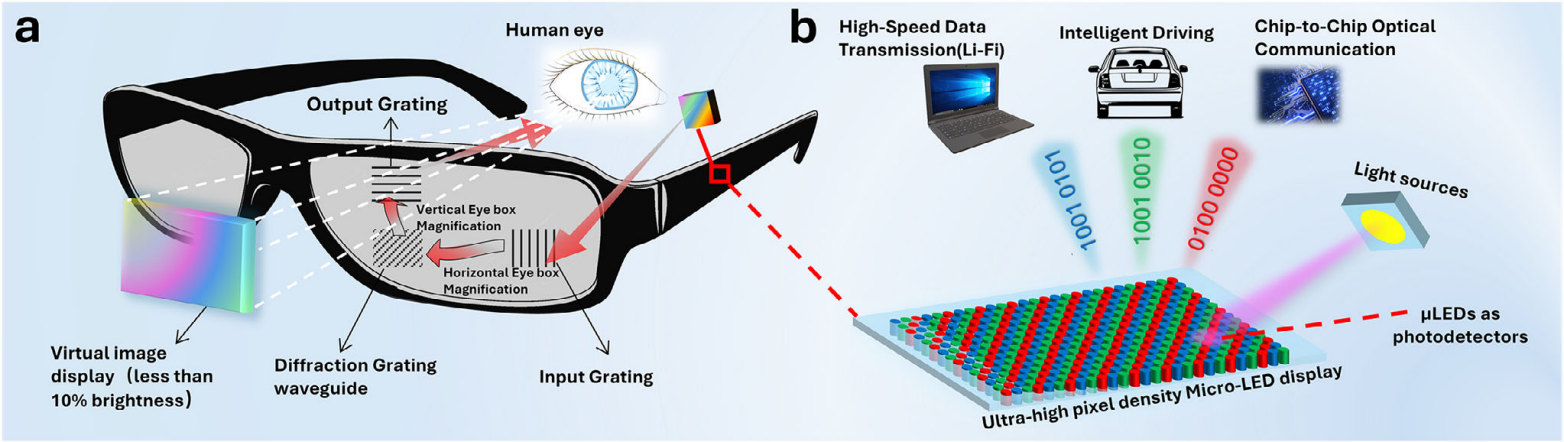
The applications of Micro‐LED in AR displays underscore the necessity of high brightness and multi‐functional integration. a) The Micro‐LED AR glasses that employ diffractive waveguides encounter significant efficiency challenges, with optical efficiencies typically falling below 1%. b) Micro‐LEDs function not only as light sources but also as high‐responsivity photodetectors for ultraviolet light and as high‐speed communication sources for visible light.

The display serves as the principal interface through which we interpret and interact with the world, with over 80% of human information acquisition occurring via visual means. The rapid progression of next‐generation information technologies, encompassing artificial intelligence, 5G/6G communications, and the Internet of Things, has facilitated an increasing integration between display technology and the processes of information perception, transmission, and processing. This convergence has given rise to a multitude of innovative application scenarios, including augmented reality (AR) / virtual reality (VR), vehicular displays, projection systems, and optical communication. These emerging applications impose specific demands on future display devices as information interfaces, necessitating attributes such as high resolution, high brightness, and multifunctional integration, as shown in **Figure** **1**. Taking AR applications as an example, considering the significant light loss that occurs during propagation through the optical module, to fully experience a seamless virtual scene with a pixel density of 60 pixels per degree and a brightness of 400 nits, it may be necessary to utilize a micro display panel with a pixel size smaller than 5 µm and a brightness exceeding 400,000 nits.^[^ ^1^, ^2^ ^]^

Among the display technologies, gallium nitride (GaN) based Micro‐LED technology offers exceptional display performances, such as high brightness, high efficiency, high pixel density, and long lifespan, and is competent in full application scenarios from small size to large size.^[^ ^3^, ^4^, ^5^, ^6^ ^]^ Additionally, it holds significant potential for multifunctional integration, including optical communication and sensing capabilities. Consequently, it is widely regarded as one of the most promising future display technologies. Nevertheless, the high‐density pixelation of Micro‐LED continues to face substantial technological challenges during pixel miniaturization using conventional mesa etching processes. These challenges encompass a decrease in external quantum efficiency (EQE) due to non‐radiative recombination in the multiple quantum wells (MQWs) active region, which is induced by sidewall damage, as well as reverse leakage current. These factors constrain the high‐brightness application scope and industrialization potential of Micro‐LED technology.^[^ ^7^, ^8^, ^9^, ^10^, ^11^ ^]^ Previous work has focused on improving the efficiency of Micro‐LEDs at low current densities through complex epitaxial processes or elaborate surface passivation techniques. Wong et al. employed atomic layer deposition (ALD) and plasma‐enhanced chemical vapor deposition (PECVD) to deposit SiO_2_ passivation layers on Micro‐LED sidewalls,^[^ ^10^ ^]^ improving chip efficiency from 24% to 33%. Similarly, Jie Bai et al. developed a novel dielectric‐template‐based selective epitaxy process to fabricate green LEDs with a peak external quantum efficiency (EQE) of 9%.^[^ ^12^ ^]^ Lee et al. innovatively proposed several sidewall passivation techniques, including the application of initiated chemical vapor deposition to fabricate poly(1,3,5‐trimethyl‐1,3,5‐trivinyl‐cyclotrisiloxane) as an organic passivation layer. This approach significantly improved the electroluminescence intensity and peak external quantum efficiency of the devices. Moreover, the researchers conducted a comparative analysis of the performance enhancement effects of three distinct passivation materials (SiO_2_, Al_2_O_3_, and Si_3_N_4_) on AlGaInP‐based red Micro‐LEDs. Their findings revealed that the use of Al_2_O_3_ as the passivation layer resulted in superior current density. Additionally, the study investigated the impact of localized surface plasmon (LSP) and sol‐gel passivation on the performance of blue Micro‐LEDs and LED arrays, demonstrating that the synergistic effect of LSP coupling and sol‐gel passivation significantly enhanced the EQE of the devices.^[^ ^13^, ^14^, ^15^ ^]^ However, research on enhancing EQE under high current densities remains scarce, a critical gap particularly for small‐sized Micro‐LEDs and Nano‐LEDs where such improvements are essential.

Ion implantation is a planar processing technique that has been widely used in patterning and doping in the semiconductor industry. It uses specific high‐energy ions to introduce lattice damage and decreasing conductivity of semiconductor material in the selective region defined by mask, thus achieving the effect of electrical isolation. Compared to the dry etching process, ion implantation offers distinct advantages in enhancing process reliability and yield. These advantages include superior patterning resolution, precisely controlled isolation levels, and planar processing without material removal. In particular, the planar processing characteristics significantly facilitate the subsequent fabrication of metal bumps and the ensuing bonding processes. Therefore, by optimizing the processing parameters, ion implantation may enable the fabrication of Micro‐LED arrays that simultaneously achieve high resolution, high efficiency, high brightness, and a high effective light‐emitting area ratio. Despite the reported fabrication of Micro‐LED arrays using ion implantation,^[^ ^16^ ^]^ a systematic investigation into their isolation effects and corresponding photoelectric performance remains insufficient.

In this study, we present the development of high‐brightness and high‐efficiency Micro‐LED arrays through precision‐selective ion implantation (PSII), utilizing precisely engineered injection doses and energies. The impact of ion implantation on electrical isolation and optoelectronic properties of Micro‐LEDs were comprehensively analyzed. By employing the PSII pixel isolation technique, we achieved Micro/Nano‐LED arrays with a pixel density of up to 25,400 ppi. These prototypes were utilized in ultraviolet photodetectors (PDs), optical communication systems, and augmented reality (AR) displays, demonstrating substantially enhanced performance compared to devices fabricated using etching methods. These high‐brightness Micro‐LED arrays based on the PSII pixelation strategy hold great potential for multifunctional integrated applications.

## 2 Results and Discussion

### 2.1 Effects of F Implantation on the Micro‐Structures and Electrical Isolation

**Figure 2:**
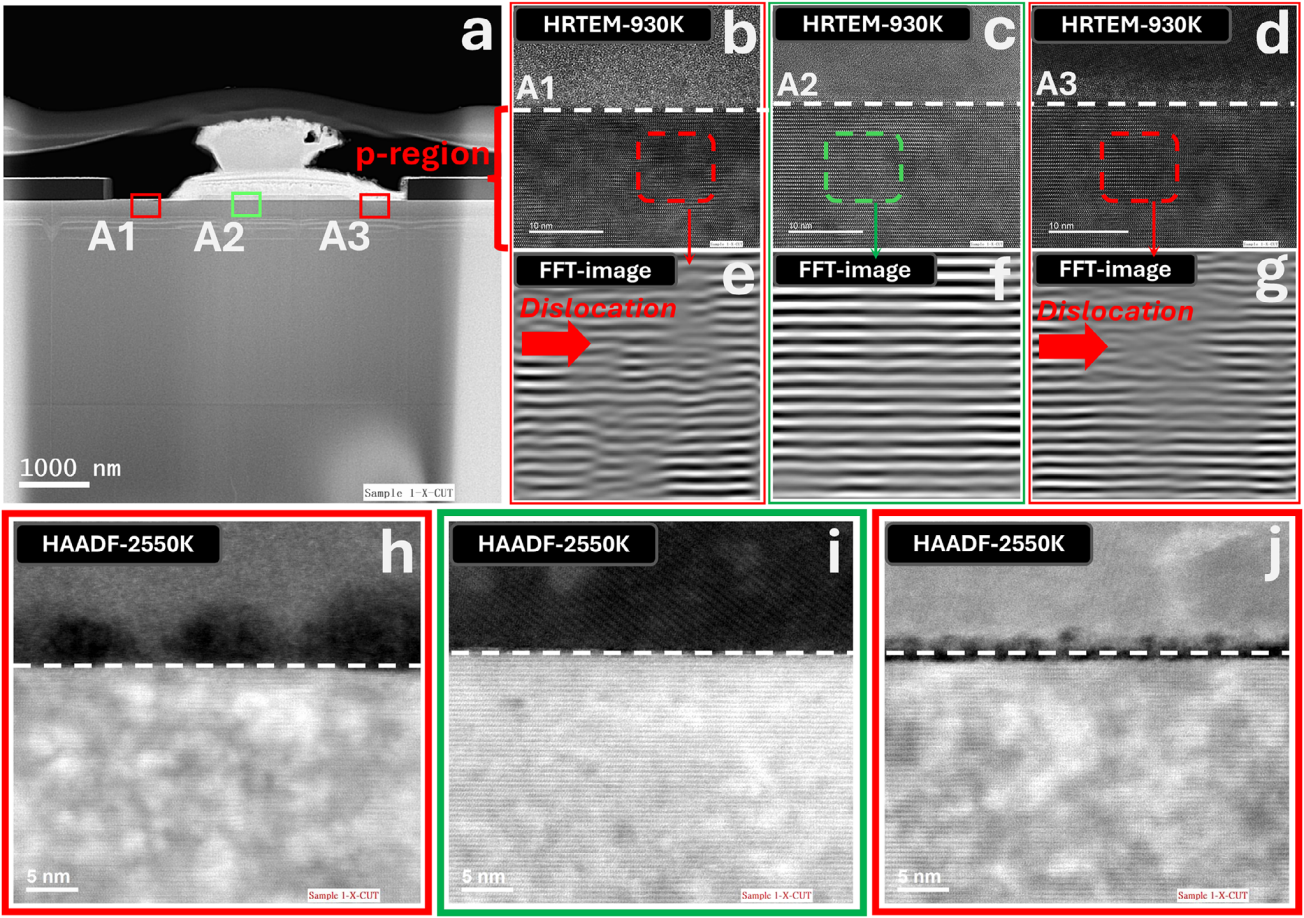
TEM images showing the lattice distortion after F ion implantation. a) Cross‐section TEM image at a single pixel. b–d) High‐magnification TEM‐HRTEM images of the injeceted area and non‐injeceted area. e–g) Fast Fourier Transform (FFT) images of the injeceted area and non‐injeceted area. h–j) High‐magnification TEM‐HAADF images of the injeceted area (A1, A3) and non‐injeceted area(A2).

Effective ion implantation significantly enhances electrical isolation, pixel definition, and light absorption, all of which are crucial for the advancement of GaN‐based optoelectronic devices. To elucidate the effects of F implantation on pixelation, we first examine the lattice damage, lattice distortion, and amorphization in the implanted p‐GaN layer using transmission electron microscopy (TEM), X‐ray photoelectron spectroscopy (XPS), and X‐ray diffraction (XRD). As depicted in **Figure** **2**, the crystal structures of p‐GaN in the implanted regions (A1 and A3) exhibit notable differences compared to the non‐implanted region (A2). High‐resolution TEM (HRTEM) images, Fast Fourier Transform (FFT) analysis, and ultra‐high‐magnification TEM‐HAADF images are presented in Figure 2b–j. HRTEM images clearly reveal the GaN lattice arrangement, demonstrating evident lattice damage and distortion in the implanted regions (A1 and A3), while region A2 (mask‐protected area) remains largely undisturbed. FFT analysis, particularly focusing on the (001) crystallographic orientation, further confirms the presence of significant dislocations in regions A1 and A3, in contrast to the well‐preserved lattice structure in region A2. Moreover, TEM‐HAADF images indicate that the F‐implanted regions exhibit a distinctly polycrystalline and amorphization characteristic.

**Figure 3:**
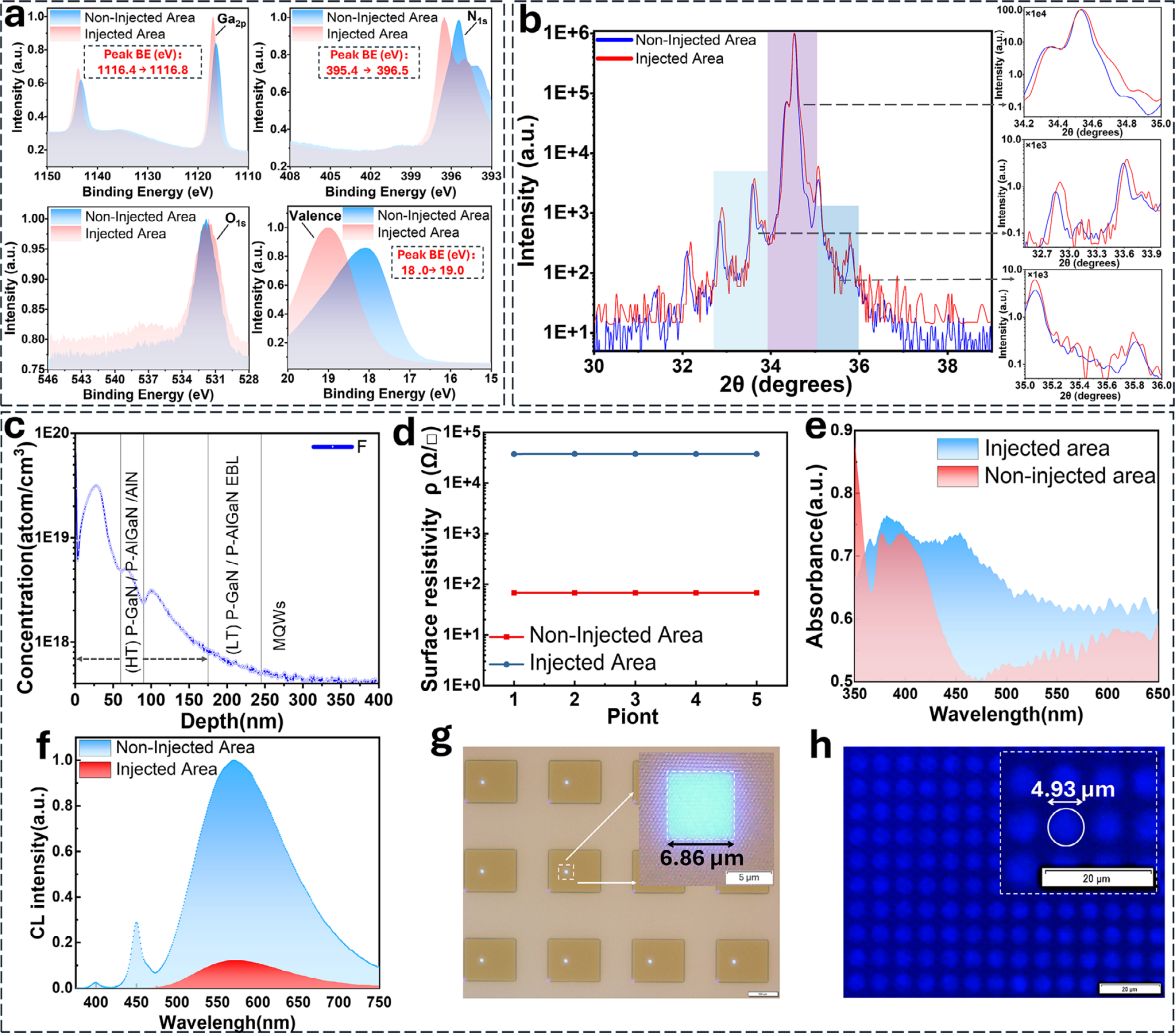
Effects of F implantation on the micro‐structures and electrical isolation. a) The Ga_2p_、N_1s_、O_1s_ and Valence XPS core‐level spectra with injected area and non‐injected area. b) XRD 2θ peaks. c) Depth distribution of F ions in the implanted InGaN LEDs. d) Electrical properties of F ions implanted InGaN LEDs, after ion injection, the average surface resistivity increased from 68 to 37,406Ω/□. e) The visible light absorbance test using a UV‐visible near‐infrared spectrophotometer for epitaxial layers with and without PSII injection. f) The fluorescence spectrum in the UV–vis band was tested using a steady‐state transient fluorescence spectrometer. g) The PL image of a Micro‐LED array with a designed square emission area of 7 × 7 µm^2^, the inset displays an enlarged image of a single pixel, showing the integrity of the pixel edges and the high effective light‐emitting area ratio. h) Luminescent display array with ultra‐high pixel density after injection isolation, the inset displays an enlarged image of pixels, showing the integrity of the pixel edges.

The lattice dislocations in the implanted p‐GaN layer were further investigated using XPS and XRD analyses. The XPS full‐survey spectra, reflecting the elemental composition of the GaN film in both the implanted and non‐implanted regions, are presented in the Supporting Information (Figure S3, Supporting Information). Additionally, high‐resolution XPS spectra of Ga_2p_, N_1s_, O_1s_, and the valence band were analyzed, as shown in **Figure** **3**a. The results indicate that the binding energies of Ga_2p_ and N_1s_ increased from 1,116.4 and 395.4 eV to 1,116.8 and 396.5 eV, respectively, after the implantation of F ions. This shift in binding energy can be attributed to the high electronegativity of F ions, which reduces the electron cloud density around neighboring atoms, thereby increasing the energy required to excite core electrons. Furthermore, a slight decrease in the O_1s_ binding energy and peak broadening were observed after F ion implantation, suggesting an increase in the proportion of chemical states associated with higher electron cloud density (e.g., hydroxyl groups ─OH), while those associated with lower electron cloud density (e.g., Ga–O bonds) decreased. Additionally, the binding energy of valence electrons increases from 18.0 to 19.0 eV following F ion injection. Due to the significantly higher electronegativity of N compared to Ga, the electron cloud is predominantly shifted toward the nitrogen atom, resulting in the formation of a polar bond. GaN tends to adsorb elements with high electronegativity, such as O and F, which can capture valence electrons within the Ga─N bond. This causes the valence electrons to move closer to the atomic nuclei of O or F, thereby substantially increasing the binding energy. Similarly, a slight broadening of peaks was observed in the XRD patterns (Figure 3b), and no shift in the AlGaN satellite peaks or the emergence of shoulder peaks was detected, suggesting that point defects—ranging from isolated point defects to defect complexes—are predominantly generated under the given implantation conditions.^[^ ^16^, ^17^ ^]^ It should be noted that the concentration of F^−^ is extremely low (implantation dose: 5 × 10^14^ atoms cm^−2^), which prevents both XPS and EDS from detecting any F‐related signals (Supporting Information Figures S2 and S3, Supporting Information). The XPS and XRD results provide indirect evidence that F ion implantation introduces lattice distortion, which might cause the electrical properties of GaN.

During the implantation, high‐energy ions implanted into the epitaxial structure may experience deviations from their initial trajectory due to lattice scattering within the target material, eventually coming to rest at a specific depth once their kinetic energy is fully dissipated. Excessive deviation can compromise the integrity of the epitaxial layers beneath the implantation mask, thereby adversely affecting the luminescence performance. The lateral mapping (Figure S4b, Supporting Information) indicates that the injection mask successfully confined F ions, with minimal diffusion beneath the mask (the black central region within the white dashed circle). Furthermore, F‐ ions were effectively implanted into the p‐GaN layer, which has a thickness of ≈245 nm. A small fraction of these ions penetrated deeper into the electron‐blocking layer and MQWs region, as evidenced by the depth profiles of the F element presented in Figure S4c (Supporting Information). As illustrated in Figure 3c, the distribution of F‐ ions in GaN is non‐uniform, exhibiting a Gaussian‐like concentration profile. Discontinuities in the distribution are also observed at the interfaces between the p‐GaN and p‐AlGaN, likely attributable to compositional differences and variations in doping levels. In this study, the selection of optimized ion implantation energy and dose for F ions was guided by simulation results obtained prior to the actual ion implantation process. Although higher implantation energy and dose can improve electrical isolation, they may also result in increased lateral diffusion, which compromises the fidelity of pixel definition and brightness (Figure S4d–f, Supporting Information). Therefore, the majority of F ions were implanted into the p‐GaN layer, with only a minimal amount penetrating into the MQW layer.

The F‐ion implantation induces a significant variation in the conductivity of GaN, thereby achieving electrical isolation. The electrical property was systematically evaluated using the four‐probe method, with multiple samples analyzed to ensure data reliability. As shown in Figure 3d, the average surface resistivity of the epitaxial layers increased dramatically from 68 Ω/□ to 37,406 Ω/□ after implantation. This substantial increase reflects a notable alteration in the electrical properties of GaN, thus confirming the high efficacy of PSII in realizing electrical isolation. The variation in electrical characteristics following ion implantation can be attributed to lattice damage induced by ion bombardment. As depicted in the visible light absorbance spectra of GaN presented in Figure 3e, light absorption is significantly enhanced after ion implantation, with a particularly notable increase of nearly 50% observed in the blue band. GaN possesses an intrinsic bandgap of ≈3.4 eV and demonstrates inherent sensitivity to ultraviolet (UV) light below 365 nm. However, lattice damage induced by high‐energy ion implantation gives rise to deep‐level defects, which result in energy band bending and bandgap narrowing (Figure S5, Supporting Information), consequently shifting the photosensitive range toward the blue wavelength region.^[^ ^18^ ^]^ This enhancement in light absorption could potentially broaden the operational range of GaN‐based photodetectors.

The fluorescence characteristics were further investigated using a steady‐state transient fluorescence spectrometer, as depicted in Figure 3f. In the UV–vis spectrum of the untreated sample, three distinct emission features are identified: a peak at ≈400 nm, which is associated with the superlattice structure introduced for thin‐film stress optimization; a peak at 450 nm, resulting from conventional photoluminescence excitation that generates blue light; and a broad yellow emission band centered at 570 nm, attributed to radiative recombination between shallow donor and deeper acceptor energy levels.^[^ ^19^ ^]^ After high‐energy ion implantation, the emission peaks at 400 and 450 nm are almost completely suppressed, highlighting the effectiveness of PSII in achieving optical isolation. Additionally, the yellow emission band at 570 nm is substantially attenuated. This reduction is primarily attributed to the inherently strong yellow luminescence observed in as‐grown GaN crystals, where ion‐induced modifications have a relatively minor impact on its intensity.^[^ ^20^ ^]^ Therefore, the retention of GaN following ion implantation would minimally impact the contrast of Micro‐LED displays, even under ultraviolet irradiation.

The influence of ion implantation on pixel definition has been validated through photoluminescence (PL) measurements, confirming superior pixel integrity and luminous uniformity (Figure S6a, Supporting Information). Figure 3g presents the PL image of a Micro‐LED array with a designed square emission area of 7 × 7 µm^2^. The actual emission area is approximately 6.86 × 6.86 µm^2^, indicating a high effective light‐emitting area ratio. Figure 3h shows the microscopic image of a PL Micro‐LED array with a circular pixel diameter of 5 µm and a pitch of 8 µm. The bright spots exhibit well‐defined pixel edges and a high effective light‐emitting area ratio, with a spot diameter of ≈4.93 µm. In contrast to traditional mesa etching, the effective light‐emitting area ratio decreases with shrinking pixel sizes due to plasma‐induced damage to the pixel sidewalls. Micro‐LEDs fabricated using PSII demonstrate nearly undamaged sidewalls, thereby maintaining sharp pixel edges and a high effective light‐emitting area ratio. This damage‐free fabrication approach overcomes scalability limitations, enabling Micro‐LEDs to achieve submicron or nanoscale dimensions. These findings validate precise control over ion distribution and substantial modification of GaN material properties achieved via optimized F‐ion injection energy and dose. These results emphasize the revolutionary potential of PSII in GaN‐based optoelectronic devices. The enhanced absorption of blue light extends the operational range of GaN photodetectors into the visible spectrum, while the suppression of critical emission peaks and a significant increase in surface resistivity highlights the technique's capacity to ensure robust optical and electrical isolation. Such advancements are pivotal for enhancing device performance in applications such as high‐resolution displays and advanced sensors.

### 2.2 Photoelectric Characteristics of Micro‐LED by Ion Implantation

**Figure 4:**
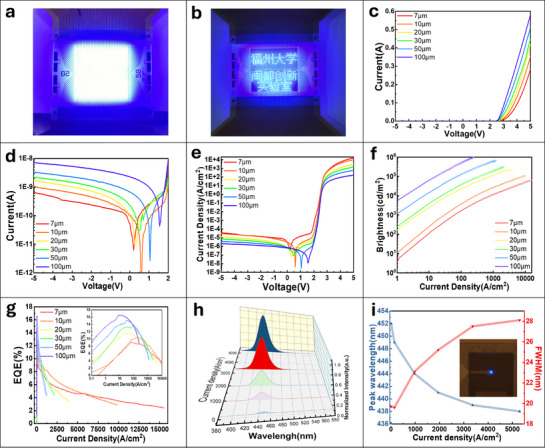
Photoelectric characteristics of Micro‐LED by ion implantation. a) Image of a full illumination image. b) Display of related Chinese characters. c,d) The current‐voltage characteristics of a Micro‐LED with respect to its emitting size. e) The current density‐voltage characteristics of an LED with respect to its emitting size. f) Current density–luminescence curves of PSII‐based Micro‐LED with various emitting size. g) The EQE as a function of current density of the Micro‐LED arrays. h) Normalized EL intensity of PSII blue Micro‐LED (from 146 to 5,299 A cm^−2^). i) Peak wavelength and FWHM shift with current density elevating to 5,299 A cm^−2^.

The photoelectric properties of Micro‐LED arrays fabricated using the PSII isolation process have been further investigated. Samples with 64 × 64 arrays and lighting spot sizes ranging from 100 to 7 µm were prepared using a 500‐nm‐thick photoresist mask under F^−^ ion implantation conditions: room temperature, 0° tilt angle, 30 keV energy, and a dose of 5 × 10^14^ cm^−2^. The devices were successfully illuminated using a passive matrix driving method. **Figure** **4**a,b presents the microscopic images of the Micro‐LED arrays with full‐screen illumination and character display, respectively, demonstrating excellent luminous uniformity among pixels. For statistical reliability, 6 × 6 pixel arrays were systematically selected from each device for *I‐V‐L* characterization, as illustrated in Figure S6c,d (Supporting Information). Figure 4c–e depicts the *I‐V* characteristics in terms of current and current density across various Micro‐LED lighting sizes. Under identical forward bias conditions, larger Micro‐LED pixels exhibit higher currents, whereas smaller pixels demonstrate greater current densities due to their reduced surface‐to‐volume ratios.^[^ ^21^, ^22^ ^]^ Additionally, it was observed that reverse leakage currents decrease with diminishing emission areas, with extremely low reverse leakage currents of 8 × 10^−9^ A recorded for Micro‐LED lighting spots of 7 µm. This underscores the effectiveness of PSII in suppressing defects and ensuring excellent electrical isolation.^[^ ^23^ ^]^ The brightness versus current density curves for various lighting spot sizes are illustrated in Figure 4f. At low current densities, efficient electron‐hole injection into the quantum wells leads to radiative recombination, resulting in a near‐linear relationship between brightness and current density across all sizes. However, at higher current densities, current crowding and defect dominance impede the increase in brightness, particularly in devices with smaller light‐emitting areas. PSII‐pixelated Micro‐LED arrays with a 100 × 100 µm^2^ light‐emitting area achieve a luminance of nearly 1000,000 cd m^−^^2^ based solely on the emissive area,^[^ ^24^ ^]^ whereas those with a 7 × 7 µm^2^ light‐emitting area reach 63,000 cd m^−^^2^, demonstrating ultrahigh brightness. The EQE was calculated according to Equation (1):

(1) $$EQE=\frac{P/h\nu }{I/e}=\frac{n_{p}}{n_{e}}\;*100\%\;$$

where *P* denotes the light output power, *h* represents Planck's constant, and *v* is calculated as *c*/*λ*, with *c* being the speed of light and *λ* indicating the emission peak wavelength achieved under a specific current density. Additionally, *I* and *e* denote the forward current and the elementary charge of an electron, respectively, while np and ne represent the number of emitted photons and injected electrons, respectively. Figure 4g illustrates the trend of EQE as a function of current density, demonstrating an initial increase at low current densities, reaching a peak before subsequently declining. Furthermore, the peak values of EQE decrease and shift toward higher current densities as the luminous area diminishes. For instance, Micro‐LED arrays with a light‐emitting area of 100 × 100 µm^2^ achieve an EQE of 16.5% at a current density of 7.95 A cm^−^^2^, whereas those with a light‐emitting area of 7 × 7 µm^2^ exhibit an EQE of 9.1% at 63.5 A cm^−^^2^. This phenomenon can be attributed to the changing proportions between the non‐radiative Shockley–Read–Hall (SRH) recombination rate and the radiative recombination rate, which result from the modification of the surface‐to‐volume ratio associated with different luminous areas. The relationships between EQE, current injection efficiency (CIE), internal quantum efficiency (IQE) and light extraction efficiency (LEE) of Micro‐LED were shown in Equations (2) and (3):^[^ ^25^, ^26^, ^27^, ^28^, ^29^ ^]^

(2) EQE=CIE∗IQE∗LEE

(3) $$IQE=\frac{R_{rad}}{R_{rad}+R_{SRH}+R_{Auger}}$$

where *R_rad_*, *R_SRH_* and *R_Auger_* represent the radiative recombination rate, SRH recombination rate and Auger recombination rate of carriers respectively. As anticipated, all EQE curves demonstrate a significant decline with further increases in current density. This occurs because at low current densities, the Auger recombination rate is relatively negligible and can be disregarded. However, as the current density rises, the non‐radiative SRH centers gradually reach saturation, leading to an increase in the Auger recombination rate. Consequently, current crowding becomes more pronounced and begins to dominate device performance, thereby effectively enhancing the proportion of non‐radiative recombination.^[^ ^28^, ^30^, ^31^ ^]^

The electroluminescence (EL) spectral characteristics of a 7 × 7 µm^2^ PSII blue Micro‐LED array were systematically investigated. Figure 4h displays the emission spectra of the sample under different current densities, indicating a blue‐shift in the central wavelength as the current density increases. Figure 4i illustrates the variations of the peak wavelength and full width at half maximum (FWHM) as functions of current density. Additionally, the inset provides an EL micrograph of the sample. The peak wavelength shifts from 452 to 438 nm as the current density increases from 10 to 5,299 A cm^−^^2^. This phenomenon can be attributed to the quantum‐confined Stark effect (QCSE), which originates from indium fluctuations within the multi‐quantum wells (MQWs). Specifically, the inherent electric field induces band tilting, while higher carrier concentrations partially screen this field, thereby reducing the emission wavelength. Meanwhile, the band‐filling and thermal effects result in an increase in the full width at half maximum (FWHM) with rising current density, which can be attributed to increased electrical resistance and heat generation.^[^ ^32^ ^]^ Specifically, the FWHM increases from 19.6 nm at a current density of 146 A cm^−^^2^ to 28.1 nm at 5,299 A cm^−^^2^. Despite this broadening effect, the FWHM remains within acceptable operational limits. It is evident that the PSII pixelation strategy offers a superior alternative to conventional mesa etching techniques, enabling the fabrication of Micro‐LEDs that are free from etching‐induced damage, highly scalable, and characterized by outstanding luminance and efficiency.

### 2.3 Comparison of PSII with Etching Methods

**Figure 5:**
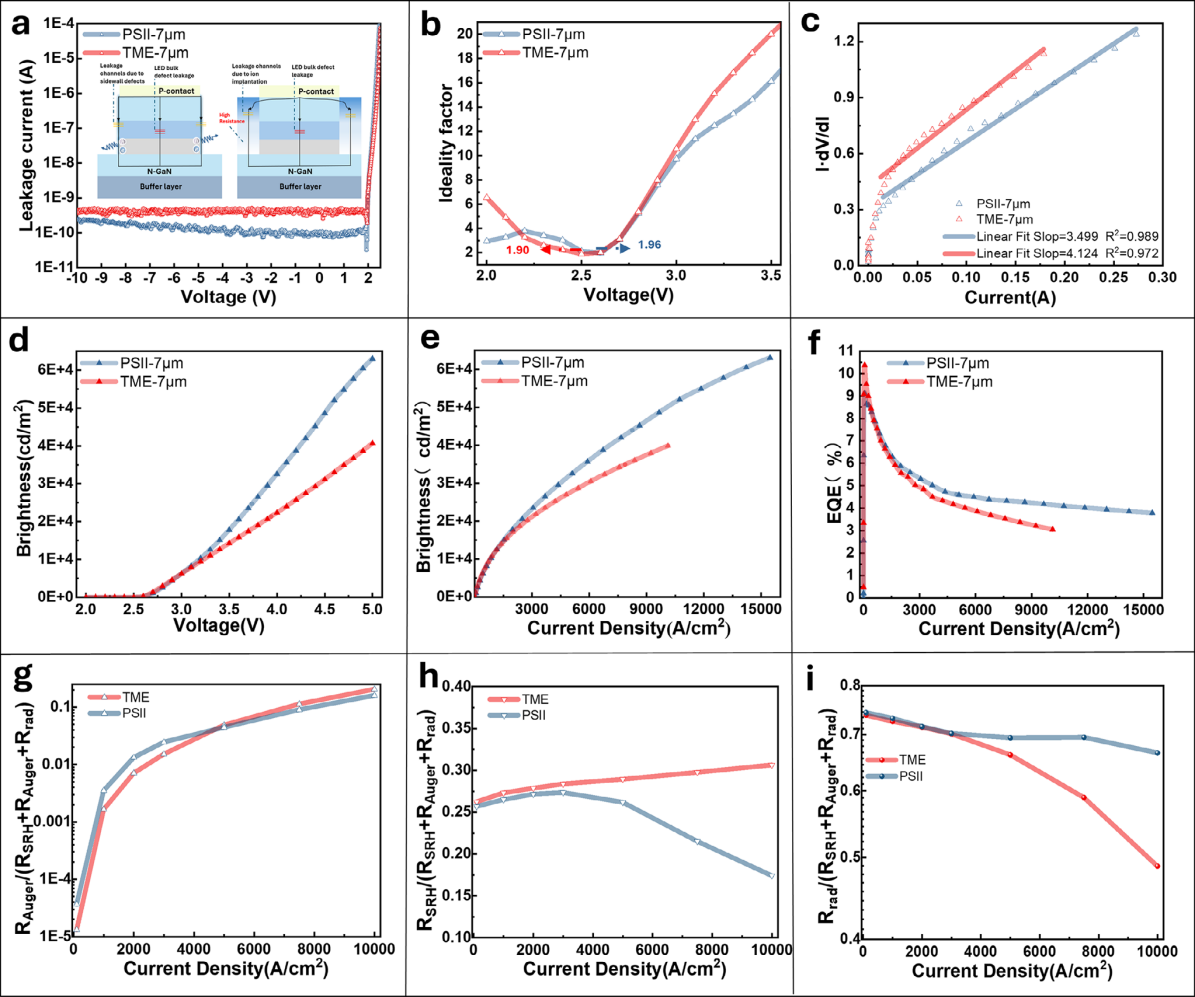
The advantages of PSII pixelation strategy. a) Leakage current at reverse bias, the inset shows the leakage current path schematic diagram in the Micro‐LED fabricated by TME and PSII. b) Ideality factor extraction within 2–3.5 V for Micro‐LEDs fabricated by TME and PSII. c) Series resistance extraction. d) The brightness versus voltage curves for 7 µm Micro‐LEDs fabricated by TME and PSII. e) The brightness versus current density curves for 7 µm Micro‐LEDs fabricated by TME and PSII. f) The EQE versus voltage curves for 7 µm Micro‐LEDs fabricated by TME and PSII. g–i) Comparative analysis of carrier recombination ratios under varying current densities between two isolation methodologies.

To further highlight the superiority of the PSII pixelization strategy compared to the traditional mesa etching (TME) method in the fabrication of Micro‐LEDs, the photoelectric characteristics of Micro‐LED arrays fabricated via ion implantation were systematically compared with those produced using TME under otherwise identical conditions, except for the pixelization technique employed. It has been demonstrated that Micro‐LED arrays fabricated using PSII exhibit a significantly lower reverse leakage current compared to those fabricated using TME, as shown in **Figure** **5**a. For instance, under a reverse bias of ‐5 V, the leakage currents of Micro‐LED arrays fabricated using PSII and TME are approximately 1.1 × 10^−10^ A and 4.5 × 10^−10^ A, respectively. This finding suggests that proper ion implantation enhances the insulation properties of the devices in the off state, thereby effectively reducing the leakage current. Additionally, in PSII‐based LEDs, leakage behavior at low forward voltages (≈0 to 2 V) has also been observed, with the leakage current being notably smaller than that of TME‐based Micro‐LEDs. This phenomenon can be attributed to the reduced number of defect‐related current paths in PSII‐fabricated Micro‐LEDs. For clarity, the leakage current paths of both PSII‐ and TME‐based Micro‐LEDs are schematically depicted in the inset of Figure 5a. These paths include bulk leakage within the LED, defect‐induced current paths caused by etching processes, and defect‐related current channels introduced by ion implantation. It is important to note that, due to the *I‐V* characteristics of LED, bulk and interface leakage currents in the LED increase substantially under forward bias conditions, coinciding with LED emission. Consequently, a higher proportion of current flows through defect regions, leading to significant non‐radiative recombination and efficiency degradation. It should also be emphasized that bulk defects in LEDs are primarily associated with material epitaxy and are largely independent of the fabrication process; therefore, they were not the primary focus of this study. Regarding the ion‐implanted regions utilized for pixel isolation, as these areas are spatially distant from the active pixels, current spreading to these regions is minimal under forward bias. In PSII‐LEDs, the high‐resistance implanted regions, which are located far from the active pixels, effectively suppress current spreading under forward bias, thereby minimizing leakage currents and reducing non‐radiative recombination.

The ideality factor (*n*), a key indicator of current transport mechanisms, was derived from the exponential *I‐V* relationships:

(4) $$I=I_{0}\;\left[\exp\left(\frac{qV}{nk_{0}T}\right)-1\right]\;$$

(5) $$n=\frac{q}{k_{0}T}\;\cdot \frac{\partial V}{\partial lnI}\;$$

where *I_0_* is the saturation current, *q* is the electron charge, *k_0_* is the Boltzmann constant, *T* is the absolute temperature.

According to Per Shockley's theory for p‐n junctions, a value of *n* ≈ 1 signifies band‐to‐band radiative recombination (diffusion current), whereas *n* ≈ 2 corresponds to Shockley‐Read‐Hall (SRH) recombination via traps (recombination current). The n‐V curves of Micro‐LED arrays with lighting areas of 7 × 7µm^2^ are presented in Figure 5b. Prior to the LED entering forward conduction, the value of *n* is relatively large, which suggests the presence of parasitic shunt resistance associated with the current. This resistance is significantly lower than the junction resistance in the equivalent circuit.^[^ ^33^ ^]^ The gradual decrease in n thereafter is primarily attributed to carrier diffusion and recombination.^[^ ^34^ ^]^ With a further increase in voltage, the effects of current crowding become predominant,^[^ ^33^, ^35^ ^]^ as the LEDs utilizing the PSII strategy can sustain higher current densities. Additionally, the absence of etching‐induced leakage channels on the pixel sidewalls contributes to suppressing the growth of the ideality factor at higher voltages.

Series resistance (Rs) was extracted from the *I·dV/dI* versus *I* relationship, as depicted in Figure 5c, with values of 3.50 Ω obtained for PSII samples and 4.12 Ω for TME devices. This discrepancy can be attributed to the high‐resistance state within the injection region. Given that the pixel and injection regions are electrically connected in parallel, this configuration leads to a reduction in the overall device resistance, thereby mitigating carrier overflow at high current densities. Notably, the linear fit used for Rs calculation in the PSII device is excellent, particularly under high current conditions.

Surprisingly, PSII‐pixelated Micro‐LEDs exhibit superior performance compared to TME counterparts in terms of EQE and luminance at high current densities. The relationships among brightness‐voltage, brightness‐current density, and EQE‐current density for 7 µm‐sized samples are illustrated in Figure 5d–f, while data for other pixel sizes are provided in Figure S7 (Supporting Information). The higher current densities at identical biases (2–5 V) for PSII‐LEDs can be attributed to their lower internal resistance and enhanced carrier injection efficiency. While the peak EQE position for the same emission area remained nearly unaffected by the two pixelization strategies, the trend of EQE reaching its peak and subsequently decaying was significantly slowed down by the PSII pixelization approach. Additionally, the luminance achieved with PSII pixelization was considerably higher than that obtained using traditional mesa etching under high current density conditions. For Micro‐LEDs with an emission size of 7 µm, the EQE was enhanced by over 30%, and the luminance was improved by nearly 25% when employing the PSII pixelation strategy at a current density of 10,000 A cm^−^^2^. Additionally, the brightness was increased by up to 54.9% (from 40,663 to 63,015 cd m^−^^2^) under a driving voltage of 5 V. It has been demonstrated that the PSII pixelation strategy can substantially improve both the display brightness and EQE at high current densities during the fabrication of small‐sized Micro‐LEDs. This advancement is crucial for enabling the application of small‐sized Micro‐LEDs in high‐brightness displays for AR glasses.

To elucidate the mechanisms underlying the performance enhancement of Micro‐LEDs using the PSII pixelation strategy, we established advanced physical models and systematically compared them with TME. The Auger recombination, SRH recombination, and radiative recombination with respect to current density for samples fabricated using the PSII and TME methods are respectively depicted in Figure 5g–i. At low carrier injection levels, the probabilities of electron‐hole collisions are minimal, rendering Auger recombination negligible (<1 × 10^−4^), while Shockley‐Read‐Hall (SRH) recombination via epitaxial defects dominates (>20%). As current density increases, Auger recombination rises exponentially, leading to the saturation of defect states within the emissive pixel. In TME‐fabricated Micro‐LEDs, excess carriers accumulate predominantly at the pixel center under high current densities, subsequently spilling over to the sidewalls where etching‐induced defects are prevalent. This phenomenon significantly elevates SRH recombination rates (e.g., reaching 30.63% at a current density of 10,000 A cm^−^^2^), thereby severely degrading EQE. Photons generated in proximity to the sidewalls either escape directly or are absorbed by surface defects, leading to reduced light extraction efficiency (LEE) and inducing pixel crosstalk that compromises display contrast. In contrast, PSII‐fabricated Micro‐LEDs effectively circumvent etching‐related sidewall damage and potential chemical contamination from volatile elements or pollution layers.^[^ ^36^, ^37^, ^38^ ^]^ Consequently, defect densities at pixel boundaries are substantially minimized, resulting in reduced SRH recombination rates (decreasing to 17.42% at 10,000 A cm^−^^2^) and enhanced radiative recombination efficiencies (increasing from 48.84% to 66.56%). The resultant improvement in internal quantum efficiency (IQE) directly contributes to a significant enhancement in EQE, as demonstrated in Equation (2). Ion implantation creates high‐resistivity GaN regions (Figure 3d) that encapsulate the emissive pixels, thereby enhancing blue‐light absorption and confining photon emission. This approach effectively minimizes sidewall photon leakage, thereby reducing crosstalk and improving contrast relative to TME, as evidenced by a high‐resolution active‐matrix PSII prototype (Figure S9, Supporting Information). The absence of etching‐induced defects further ensures the preservation of LEE, thereby amplifying overall optical performance. These findings demonstrate that PSII enhances Micro‐LED efficiency by mitigating sidewall recombination losses and optimizing carrier dynamics. The strategy's capacity to suppress non‐radiative recombination, improve IQE, and enhance optical confinement highlights its superiority over TME, providing a robust solution for high‐performance, high pixel density Micro‐LEDs in advanced display technologies.

### 2.4 Verification of AR Display and Competence of Nano‐LED

**Figure 6:**
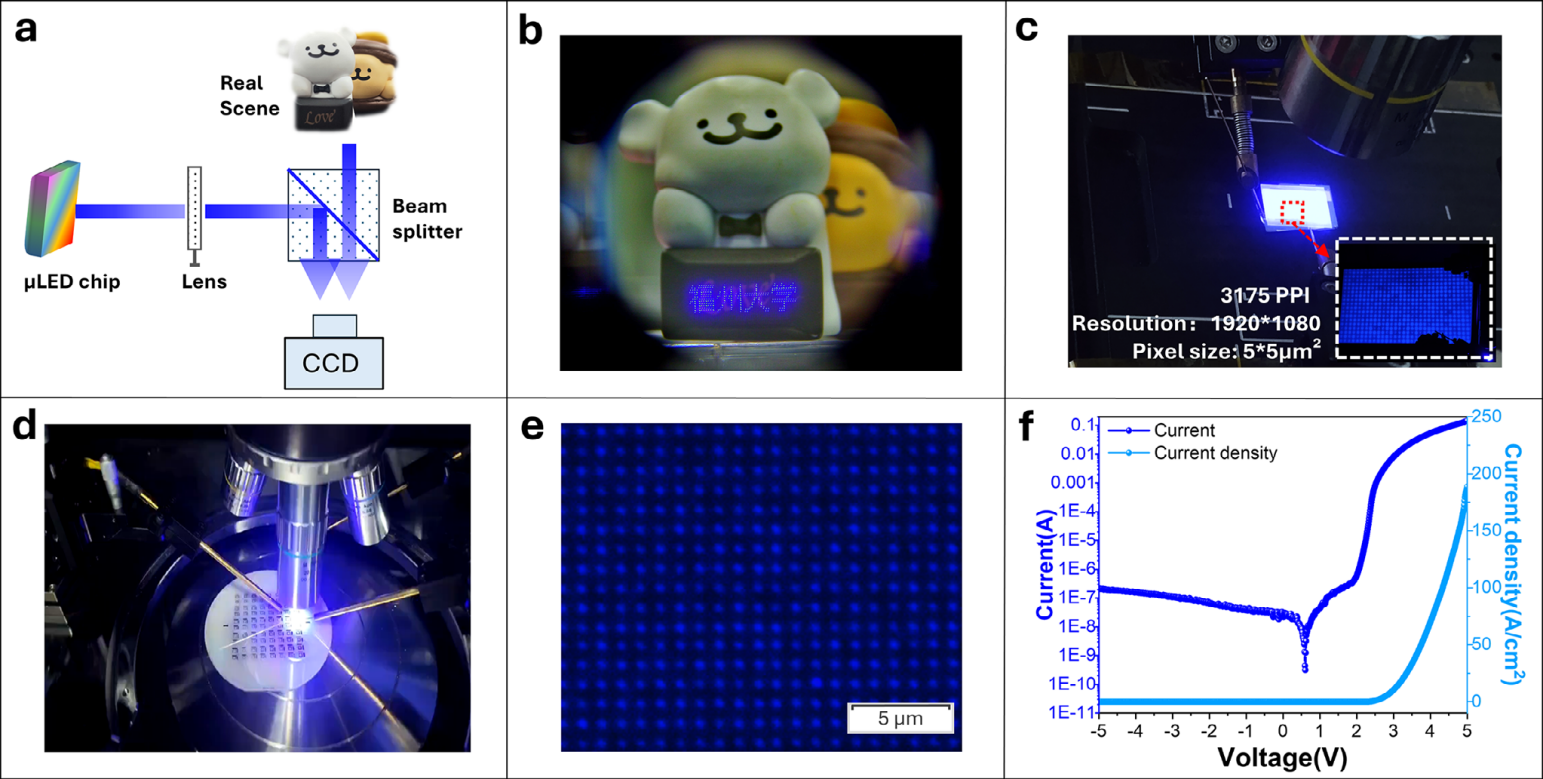
Application demonstration of Nano‐LED and AR displays. a) Schematic diagram of the principle of demonstrating the imaging effect of AR glasses using PSII‐LED as the light source. b) Demonstration of AR glasses effects, the floating virtual images of Chinese characters are displayed in front of the dolls. c) An active‐matrix‐driven ultra‐high‐resolution display prototype through metal flip‐chip bonding integration of the Micro‐LED display array with a silicon‐based driver chip. d) The Nano‐LED light‐emitting array was tested for illumination using a semiconductor probe tester. e) Microscopic observation image of the pixels of the Nano‐LED arrays with 25,400 PPI. f) The typical *I‐V* characteristic curve of the Nano‐LED arrays.

The high‐brightness Micro‐LED arrays fabricated via the PSII process have enabled a practical augmented reality (AR) application. As shown in **Figure** **6**a, our AR display prototype was developed on a high‐precision optical platform, consisting of Micro‐LED arrays, a lens, and a beam splitter. The emitted image was focused by the optical lenses, subsequently transmitted through the beam splitter, and finally projected onto the retina to generate a virtual scene. In this experiment, a CCD camera was employed to simulate the eyeball model. Figure 6b illustrates the floating virtual images of Chinese characters in front of two dolls, demonstrating clear character display. However, it is evident that floating characters exhibit relatively high graininess due to insufficient pixel density. This leads to a lack of clarity, akin to viewing through a screen, which is referred to as the screen door effect. Based on the PSII pixelation strategy, we successfully developed an active‐matrix‐driven ultra‐high‐resolution display prototype by integrating a Micro‐LED display array with a silicon‐based driver chip through metal flip‐chip bonding, as illustrated in Figure 6c. Although the figure displays non‐uniform luminescence across the pixels, this phenomenon cannot be attributed to ion implantation. Instead, it is primarily caused by epitaxial warpage or variations in the height of metal bumps, both of which negatively affect bump bonding performance. As a result, the current distribution becomes uneven, leading to a decrease in luminescence uniformity across the pixels. This prototype achieves a pixel density of 3,175 PPI, with an emissive pixel diameter of 5 µm. A comparative analysis with conventional mesa etching techniques demonstrates a significant reduction in side‐wall light leakage, thereby enhancing the display contrast ratio (Figure S9, Supporting Information). These results validate the technological superiority and feasibility of our integration approach.

For AR displays, progressively achieving higher resolutions in display devices is an effective approach to enhancing image quality and enabling near‐realistic visual experiences. As a result, the growing demand for higher resolution has led to the development of Nano‐LED displays, which feature pixels at the submicron or nanoscale level. PSII has been demonstrated to be highly effective for the preparation of Nano‐LEDs. By precisely controlling the injection energy and dose, the lateral spreading of F ions was effectively suppressed, enabling the successful fabrication of Nano‐LED arrays with a pixel density of 25,400 pixels per inch (PPI) and a resolution of 640 × 480, as illustrated in Figure 6d,e. High‐magnification microscopy confirmed exceptional light emission from individual pixels, each exhibiting a diameter of 500 nm and a pitch of 1 µm. This indicates superior uniformity and pixel integrity compared to conventional mesa etching techniques. As depicted in Figure 6f, the typical current‐voltage (I‐V) characteristic curve further substantiates excellent electrical performance. The ultra‐high density and resolution achieved in this Nano‐LED array, enabled by the PSII‐based pixelation strategy, surpasses the limitations of traditional fabrication methods, offering critical support for advanced display technologies such as future near‐eye displays and light field displays.

### 2.5 Optical Communication Performances

**Figure 7:**
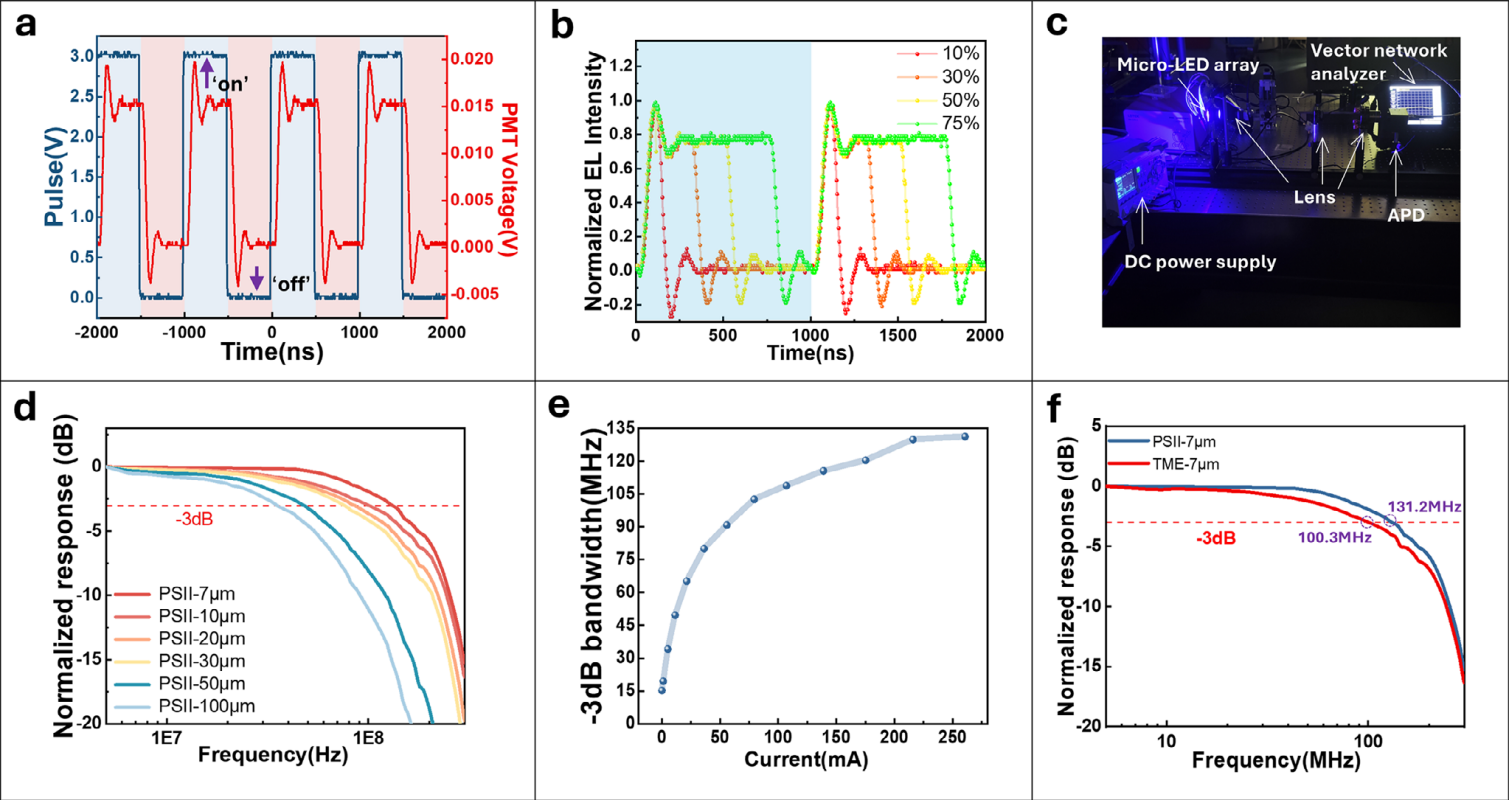
Application of Micro‐LED display arrays in optical communication field. a) Temporal response of the device in response to a square‐wave pulse bias (magnitude: 3 V, frequency: 1 MHz, and duty: 50%). b) The normalized EL intensity of the Micro‐LED in 1,000 ns, as a function of duty cycle under the 1 MHz pulse operation. c) Photograph of line‐of‐sight optical wireless communication measurements platform. d) −3 dB frequency responses from both pixel‐size Micro‐LED under the condition that both LEDs were operating at a constant DC bias of 260 mA. e) −3 dB bandwidth as a function of different current conditions. f) Frequency response measured using PSII and TME strategy.

Besides efficient electroluminescence (EL), rapid response times are essential for Micro‐LED applications in optical wireless communication (OWC) and signal modulation. This study investigates the temporal response of a 6 × 6 Micro‐LED pixel array (pixel size: 7 µm) using a high‐sensitivity photomultiplier tube (PMT) to measure EL intensity. As shown in **Figure** **7**a, the response to a square‐wave pulse bias (3 V, 1 MHz, 50% duty cycle) is characterized by rise and fall times, defined as the duration between 10% and 90% of the maximum PMT voltage. The measured values are τ_Rise_ ≈ 38 ns and τ_Fall_ ≈ 36 ns, respectively. These nanosecond‐scale responses demonstrate the Micro‐LED's capability to track high‐frequency signals with minimal latency, thereby validating its suitability for parallel data transmission in OWC systems. Figure 7b presents the normalized EL intensity as a function of duty cycle. It demonstrates that higher duty ratios prolong the on‐state duration, which in turn enhances the time‐integrated EL intensity and consequently increases the equivalent brightness.

We investigated the use of a Micro‐LED array as a transmitter in a line‐of‐sight optical wireless communication (OWC) system,^[^ ^39^, ^40^ ^]^ as depicted in Figure 7c. Frequency‐modulated signals, generated by a vector network analyzer (VNA) and combined with a DC bias using a bias tee circuit, were transmitted via the array. A silicon avalanche photodetector (APD) served as the receiver and was connected to the VNA to measure frequency responses. Figure 7d illustrates the ‐3 dB bandwidth for Micro‐LEDs with varying pixel sizes under a constant DC bias of 260 mA. The observed increase in bandwidth is attributed to the reduced pixel size, which enables higher current densities and accelerates carrier generation and recombination processes.^[^ ^41^, ^42^ ^]^ Figure 7e further elucidates the relationship between bandwidth and injection current, demonstrating that bandwidth increases as the injection current rises. From Figure 7f, the bandwidths of Micro‐LEDs fabricated using PSII and etching methods are approximately 131.2 and 100.3 MHz, respectively, indicating a greater than 30% improvement in the former. This enhancement can be ascribed to the shorter carrier lifetime and more efficient charge injection in PSII‐LEDs. Collectively, these findings underscore the promising potential of PSII‐LEDs for high‐speed optical wireless communication (OWC).

### 2.6 Photodetection Performances

**Figure 8:**
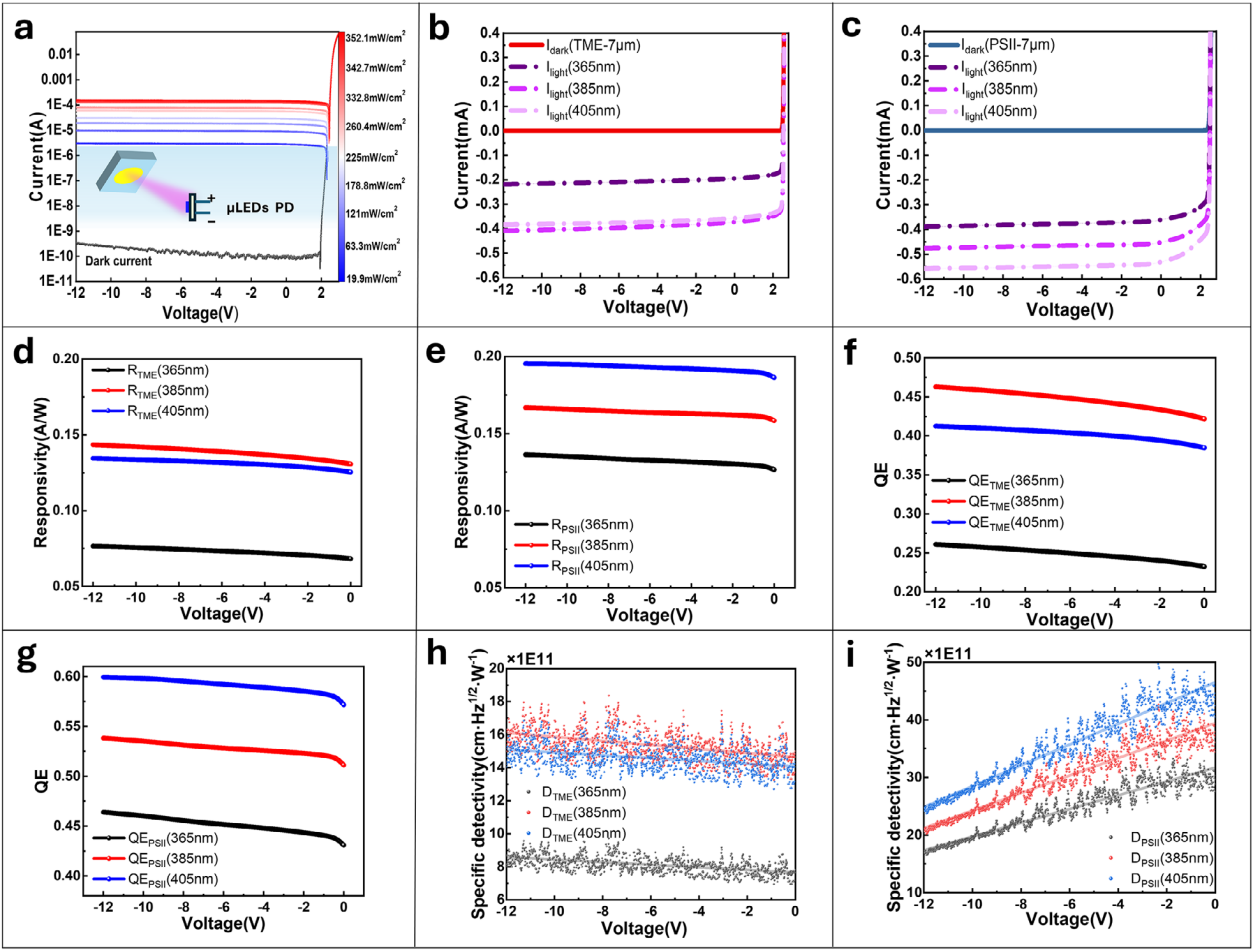
Micro‐LED arrays as photodetectors in optical communication. a) Comparison of photocurrent and dark current of PD under different light intensities. b,c) Comparison of photocurrent of PD under ultraviolet light excitation with different wavelengths for two isolation schemes. d,e) Comparison of photoresponsivity of PD under ultraviolet light excitation with different wavelengths for two isolation schemes. f,g) Comparison of photoelectric conversion efficiency of PD under ultraviolet light excitation for two isolation schemes. h,i) Comparison of specific detection rate of PD under ultraviolet light excitation for two isolation schemes.

The enhancement of UV light absorption for PSII‐based Micro‐LEDs extends the potential operational range of GaN‐based photodetectors. Additionally, we evaluated both PSII‐LEDs and TME‐LEDs as photodetector (PD) transistors under UV excitation at wavelengths of 365, 385, and 405 nm from DC‐loaded diodes. In PD mode, photogenerated carriers separate under the influence of the internal electric field, with electrons migrating to the n‐GaN region and holes migrating to the p‐GaN region, thereby generating photocurrents. **Figure** **8**a illustrates the comparison of photocurrent and dark current across various light intensities, revealing a dark current of ≈10^−10^ A at zero bias and a photocurrent exceeding 10^−4^ A at an irradiance of 352 mW cm^−^^2^, which is attributed to the increased carrier generation at higher power densities. Typical *I‐V* curves (Figure 8b,c) under dark conditions and UV illumination (352 mW cm^−^^2^) demonstrate that PSII‐LEDs exhibit superior photocurrent response compared to TME‐LEDs across all tested wavelengths, indicating enhanced optical energy collection and photovoltaic efficiency.

Responsivity (*R*), quantum efficiency (QE), and specific detectivity (D*) were assessed to quantitatively evaluate the performance of the PD. Responsivity, which reflects the photoelectric conversion capability, and quantum efficiency, which indicates the photon‐to‐electron conversion efficiency, are calculated as follows:

(6) $$R=\frac{I_{light}}{L_{in}}=\eta \cdot \frac{q\lambda }{hc}\;$$

where *I_light_* is the photocurrent, *L_in_* is the incident power, *η* is the QE, *q* is the electron charge (1.6 × 10^−19^ C), *λ* is the wavelength, *h* is the Planck's constant (6.626 × 10^−34^ J·s), and *c* is the speed of light (3 × 10⁸ m s^−1^). Figure 8d–g demonstrates that *R* decreases with reduced reverse bias due to a weaker carrier‐separating field. However, PSII‐based PDs consistently exhibit higher *R* (e.g., under ‐12 V: 0.136 A W^−1^ vs 0.076 A W^−1^ at 365 nm; 0.196 A W^−1^ vs 0.134 A W^−1^ at 405 nm) and QE (e.g., 60.0% vs 41.2% at 405 nm). The specific detectivity is defined as follows:

(7) $$D^{*}=R\cdot \sqrt{\frac{A_{\mu LED}}{2qi_{d}}}\;$$

where *A_µLED_* is the PD area and *i_d_* is the dark current. The peak values of D* were calculated as 1.69 × 10^1^ ^2^, 2.07 × 10^1^ ^2^, and 2.43 × 10^1^ ^2^ cm·Hz^1/2^·W^−1^ for PSII‐LEDs at 365, 385, and 405 nm, respectively, versus 8.58 × 10^11^, 1.61 × 10^12^, and 1.51 × 10^1^ ^2^ cm·Hz^1/2^·W^−1^ for TME‐LEDs (Figure 8h,i). Notably, at zero bias, the lower dark current of PSII‐LEDs enhances D*, which contrasts with the behavior of TME‐LEDs at ‐12 V. The nanosecond‐scale response time and improved modulation bandwidth of PSII‐LEDs demonstrate their suitability for high‐speed OWC. Additionally, their superior responsivity, QE, and detectivity as photodetectors (PDs) emphasize their potential for photodetection applications. These advancements establish PSII‐LEDs as a versatile platform for next‐generation multifunctional optoelectronic technologies.

## 3 Conclusion

In this paper, a precision‐selective ion implantation (PSII) strategy for Micro‐LED pixelation was developed. The impact of ion implantation on electrical isolation and photoelectric characteristics of Micro‐LEDs has been systematically examined. The external quantum efficiency and brightness of PSII‐based Micro‐LED arrays operated at high current densities have been found to be remarkably improved compared to those fabricated using traditional mesa‐etching (TME) method. We use PSII to achieve Micro‐LED arrays with lower device off‐leakage current, improved carrier injection efficiency, reduced non‐radiation composite ratio under high current density, thus the external quantum efficiency and luminance of PSII‐based Micro‐LEDs are improved. Compared with the TME method, PSII offers several distinct advantages. First, PSII is a well‐established semiconductor process characterized by high reliability and efficiency. Second, by precisely controlling ion implantation energy and dose, the lateral diffusion of implanted ions can be effectively regulated, enabling high‐resolution pixelation at the sub‐micron or even nanometer scale. Notably, PSII involves no material etching and employs a planarization process, which facilitates subsequent metal bump preparation and metal bonding interconnection processes. Lastly, as verified in this work, PSII significantly reduces side‐wall damage, minimizes non‐radiative recombination at high current densities, and enhances both EQE and brightness. Therefore, the PSII‐based Micro‐LEDs are essential for applications that demand high resolution and high brightness, such as augmented reality (AR) displays and optical communication systems. In this work, we successfully developed a prototype of an active‐matrix‐driven Micro‐LED display with a resolution of 1920 × 1080 and a pixel pitch of 8 µm, as well as Micro‐/Nano‐LED arrays featuring a pixel density of up to 25,400 ppi, utilizing the PSII pixel isolation strategy. For demonstration purposes, Micro‐LED arrays fabricated via the PSII method were simultaneously employed in augmented reality (AR) displays, optical communication systems, and ultraviolet photodetectors (PDs), showcasing significantly enhanced performance compared to those produced using conventional etching processes. Specifically, AR displays exhibited markedly improved brightness and contrast, while the switching speed and current tolerance in optical wireless communication (OWC) systems were also enhanced. Furthermore, ion bombardment‐induced lattice damage effectively increased UV and blue light absorption while suppressing visible emission, thereby benefiting photodetector performance. Notably, the modulation bandwidth achieved up to 131.2 MHz, representing an improvement exceeding 30%, and the photocurrent of PDs increased by ≈90% compared to etched Micro‐LEDs under identical conditions. These high‐brightness Micro‐LED arrays, enabled by the PSII pixelation strategy, demonstrate significant potential for multifunctional integrated applications.

## 4 Experimental Section

### Epitaxial Growth of GaN Epilayers

The GaN‐based epitaxial structures were grown using metal‐organic chemical vapor deposition (MOCVD). Trimethylaluminum, trimethylgallium, and ammonia (NH_3_) served as the precursor reactants. Initially, a 1.8 µm thick unintentionally doped GaN buffer layer was deposited on the patterned sapphire substrate. The epitaxial layers were sequentially deposited in the following order: a 1.8‐µm‐thick Si‐doped n‐GaN layer, a 30 nm Si‐doped n‐Al_0.05_GaN electron spreading layer (ESL), a stress‐relaxation layer (SRL‐18 nm) comprising of u‐In_0.05_GaN/n‐GaN, a 83 nm Mid‐GaN layer, a 180 nm MQWs for blue emission, an electron blocking layer (EBL) composed of 18 nm GaN and 7 nm AlGaN, a 45 nm Mg‐doped low temperature (LT) p‐GaN layer, and a 175 nm Mg‐doped high temperature (HT) p‐GaN layer and p‐AlGaN/AlN.

### Device Fabrication Process

To improve the current spreading, an initial deposition of a 75‐nm indium tin oxide (ITO) layer onto p‐GaN was conducted using electron‐beam evaporation technology. Subsequently, the ITO layer was subjected to annealing at 500 °C for 20 min in an oxygen‐rich ambient atmosphere to enhance its transparency. For the fabrication of 25,400 PPI Nano‐LED arrays, maskless laser direct writing technology was employed to achieve patterning. This process resulted in a photoresist array featuring a thickness of ≈800 nm, a diameter of 500 nm, and a periodicity of 1 µm. The obtained photoresist array was subsequently utilized as a mask for ion implantation. Thereafter, patterned photoresist was utilized to define the luminous pixels, and the exposed ITO regions were selectively etched away using a mixture of HCl and FeCl_3_. Thereafter, F ions were implanted into the exposed p‐GaN utilizing the GSD/200E^2^ high current ion implanter from Axcelis to achieve inter‐pixel electrical isolation, as shown in Figures S1 and S4a (Supporting Information). The mask‐protected region was designed to emit compound luminescence without interference. Following this, the photoresist mask was stripped. Inductively coupled plasma (ICP) etching with BCl_3_/Cl_2_ = 5/25 SCCM was utilized for p‐GaN to etch p‐type regions and multiple quantum wells, exposing the common n‐GaN. It is worth noting that the etching process was not intended for pixel definition and the etched region was far away from the pixels, thus causing no damage to them. Subsequently, p/n‐electrodes (From the bottom to the top are Cr‐30 Å /AlCu‐1,000 Å /Ti‐600 Å /Pt‐500 Å /Ti‐600 Å /Pt‐500 Å / Ti‐600 Å /Pt‐500 Å /Au‐18,000 Å, respectively.) were patterned on the samples through a standard lift‐off process using negative photoresist, followed by 10 min annealing at 250 °C in N_2_. Finally, the sapphire substrate was thinned to 280 µm by chemical mechanical polishing (CMP), and the wafer was diced into six dice. Each die, consisting of a Micro‐LED array with a specific luminescence size, was connected to a pre‐developed peripheral circuit via an FPC.

### Testing and Characterization

All the characterizations of photoelectric properties were conducted at room temperature. The distribution of implanted ions was investigated by dynamic secondary ion mass spectrometry (20LA‐4570, ULVAC‐PHI, Inc.) and time‐of‐flight secondary ion mass spectrometry (PHI nanoTOF 3, ULVAC‐PHI, Inc.). The absorption rate of the material was measured by a Cary 7000 UV‐Vis‐NIR spectrophotometer from Agilent, USA. The photoluminescence spectra were tested by a FLS1000 steady‐state and time‐resolved fluorescence spectrometer from Edinburgh Instruments, UK. The surface resistivity was measured by a TH2515 resistance meter from Tonghui. Using the multi‐functional integrated LED test system (D3000‐16CH) consisting of a high‐pressure source measurement unit and a TOPCON integrated spectral radiometer SR‐3A, synchronized current‐voltage‐luminance (I‐V‐L) measurements were performed on Micro‐LED arrays using a multifunctional D3000‐16CH LED test system, integrating a high‐pressure source measurement unit and a TOPCON SR‐3A spectral radiometer. A high‐sensitivity photomultiplier tube (PMT) was utilized to detect the EL intensity and investigate the temporal response characteristics of 6 × 6 Micro‐LED pixel array device (pixel size: 7 µm). A measurement system composed of a microwave vector network analyzer (Ceyear 4957D), a current source (Keithley, 2614B), a bias circuit (Mini‐Circuits ZFBT‐6GW+, 6,000 MHz), an avalanche photodiode (Thorlabs APD 210), and a lens was employed to study the optical communication performance of the Micro‐LED array. The Micro‐LED was used as an APD, and the responsivity, photoelectric conversion efficiency, and specific detection rate of the Micro‐LED‐based PD were measured using commercial LEDs with wavelengths of 365, 385, and 405 nm. The content displayed by the Micro‐LED chip was fused with the real scene through a beam splitter and lens optical path, demonstrating its application prospects as AR‐glasses.

## Conflict of Interest

The authors declare no conflict of interest.

## Author Contributions

J.Y.Y. and X.T.Z. conceived the project. J.Y.Y. was responsible for device fabrication, data acquisition and analysis, device mechanism analysis, algorithm design, and manuscript writing. W.J.S. was responsible for device fabrication, data acquisition and analysis. Y.B.L. and Y.Y.P. were responsible for device fabrication and algorithm verification. Y.A.Z., C.X.W., J.D.Y., J.S., Q.Y., and T.L.G. were responsible for overseeing the overall work. X.T.Z. supervised the project. All authors contributed to the data analysis and approved the final manuscript.

## Supporting information



Supporting Information

## Data Availability

The data that support the findings of this study are available from the corresponding author upon reasonable request.
