# Supplementary material for: High‐Performance Micro‐LED Displays via Etching‐Damage‐Free Pixelation Strategy for Multifunctional Integrated Applications
Source: Adv Sci (Weinh). 2025 Sep 3;12(44):e11520. doi: 10.1002/advs.202511520 (PMC12667502; doi:10.1002/advs.202511520)
Supplement: Supplementary file 1 — Supporting Information [file ADVS-12-e11520-s001.pdf]

Supporting Information

High-Performance Micro-LED displays via Etching-Damage-Free pixelation strategy for multifunctional integrated applications

Jinyu Ye [1], Wenjuan Su [1], Yibin Lin [2], Yuyan Peng [1], Xiongtu Zhou [1,2,], Tailiang Guo [1,2], Jiade Yuan [1], Jie Sun[1,2], Qun Yan[1,2], Yongai Zhang [1,2,*], Chaoxing Wu [1,2,*]*

[1] College of Physics and Information Engineering, Fuzhou University, Fuzhou 350108, P.R. China.

[2] Fujian Science & Technology Innovation Laboratory for Optoelectronic Information of China, Fuzhou 350108, P.R. China.

[*]**Corresponding authors**: xtzhou@fzu.edu.cn (X.T. Zhou), yongaizhang@fzu.edu.cn (Y.A. Zhang), chaoxing_wu@fzu.edu.cn (C.X. Wu)

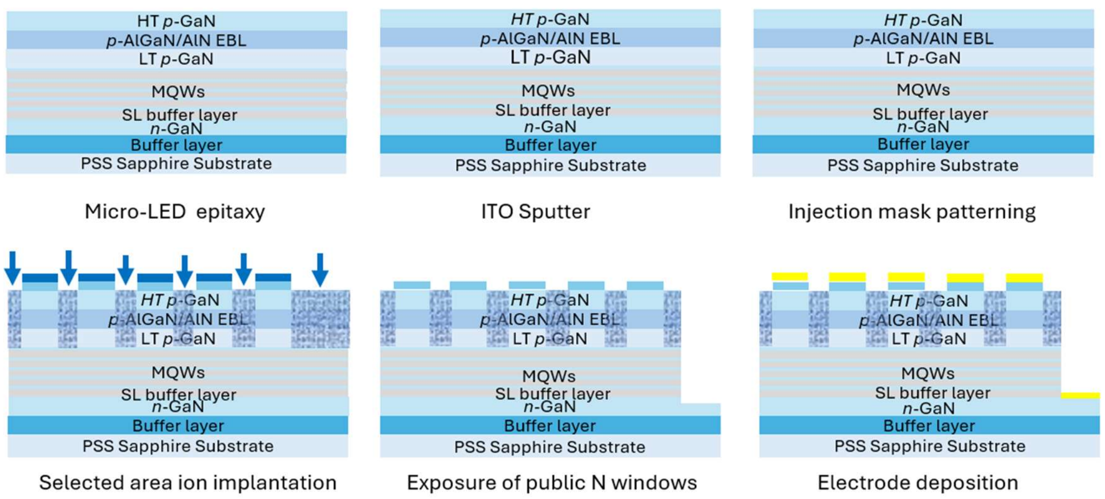

Figure S1. The process flows PSII-LED.

We selected a high-density Micro-LED array with a pixel pitch of 8 μm as the test sample. Figure S2a shows its SEM micrograph, where a single pixel and the regions on both sides were chosen as the FIB section area. A low-magnification TEM-HAADF image was taken to capture the entire sectioned region, as presented in Figure S2b. The ion-implanted edge was selected as the EDS observation area, and its HAADF image along with the elemental distribution are shown in Figures S2c–f. Similarly, EDS analysis was performed on the MQW region adjacent to the p-GaN, as depicted in Figures S2g–l.

It is known that STEM has a detection limit for the concentration of observed elements, typically ranging from 0.1% to 1%. In our work, the maximum concentration of F ions in the epitaxial layer is only approximately 3×10^{19} atoms/cm^3, while the atomic density of gallium nitride is about 8.77×10^{22} atoms/cm^3. The concentration of F ions is far below the detection limit of STEM; therefore, the signal detected in Figure S2f is actually a noise signal and has no reference value. This is further confirmed by the absence of characteristic peaks of F element in Figure S2m. Thus, it is relatively challenging to verify the absence of F residues near the p-GaN layer using TEM-HAADF. The F element distribution studied by SIMS in the main text of our paper also confirms that there is a small amount of F element near the p-GaN.

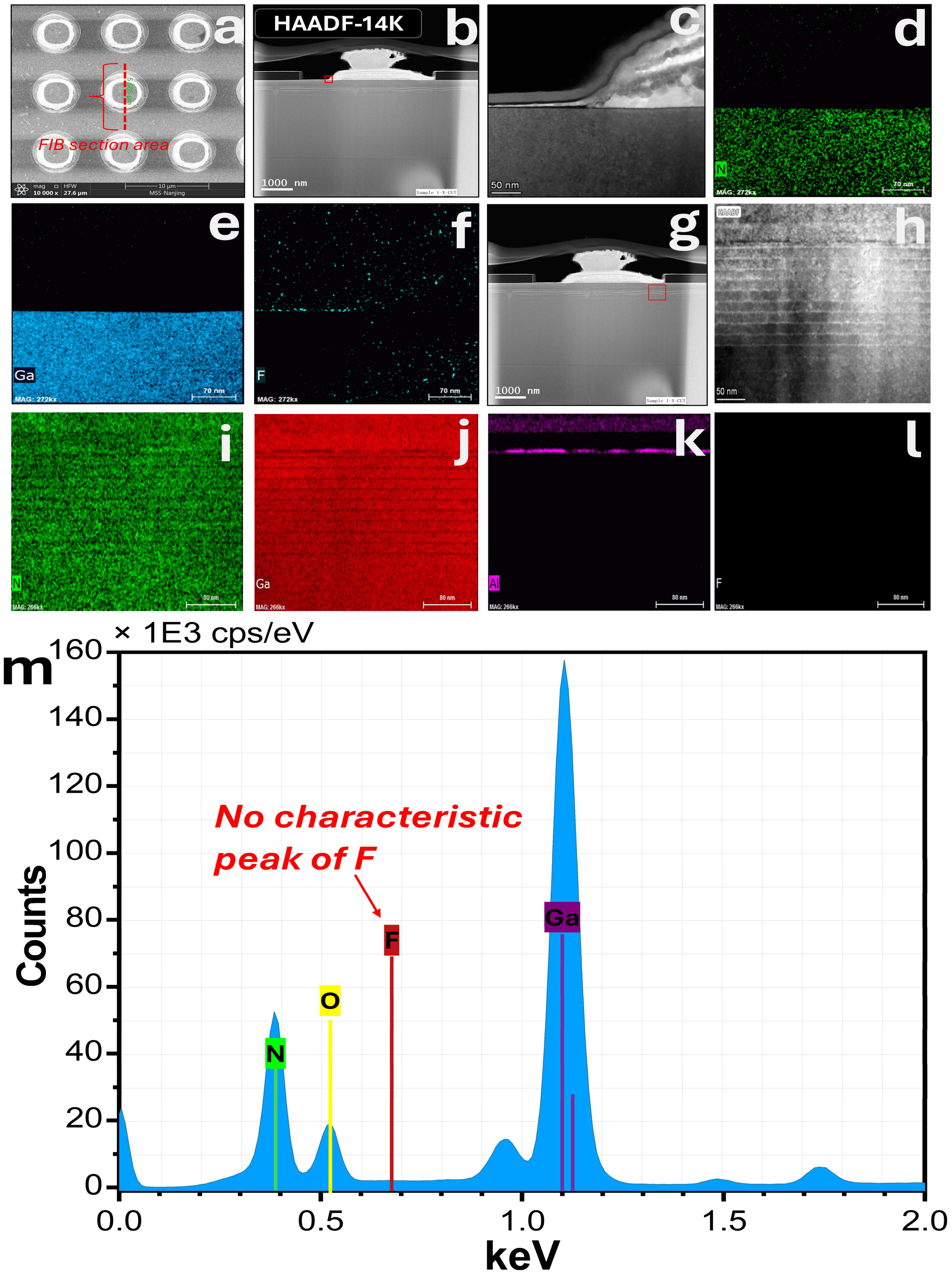

Figure S2. The SEM/STEM and their corresponding EDS images captured for the p-GaN and MQWs regions.

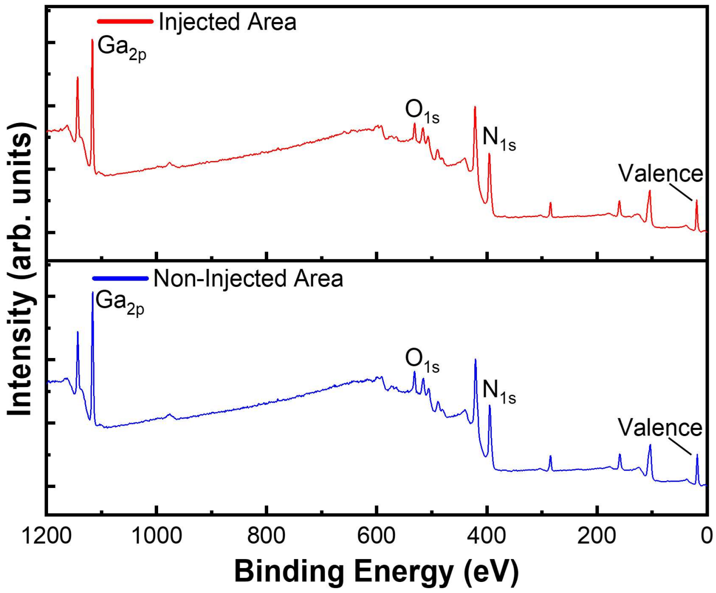

Figure S3. The XPS survey scan with injected area and non-injected area.

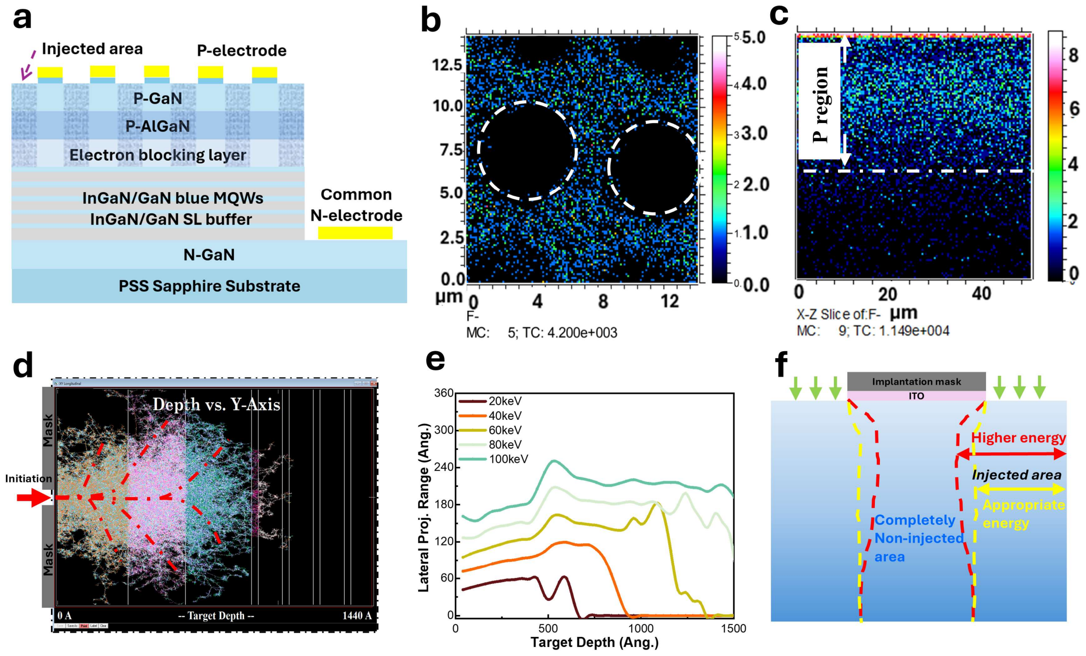

Figure S4. a) Schematic diagram of ion implantation isolation for pixelation of Micro-LED arrays. b) Lateral spreading of F ions in the implanted InGaN LEDs. c) Depth distribution of F ions in the implanted InGaN LEDs. d–f) Simulation analysis of the influence of the degree of lattice damage on the brightness and fidelity of pixel definition.

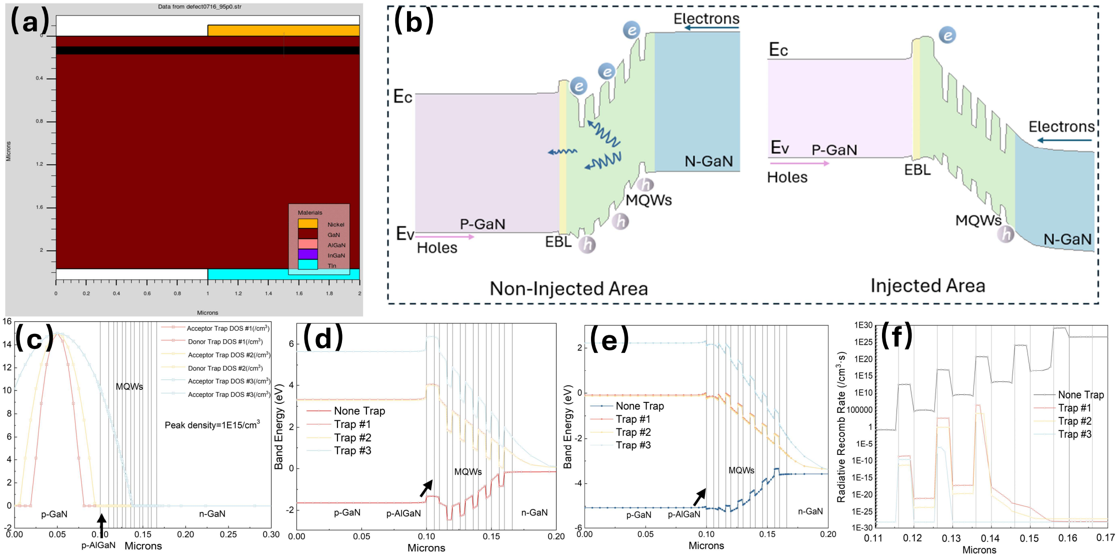

Figure S5. a) Silvaco TCAD simulation of the device structure diagram of ion implanted Micro-LED. b) Ion injection causes energy band bending and narrowing of the bandgap. c) Concentration distribution of acceptor and donor defects in the injection region under three ion distribution conditions. d–e) Comparison of energy band bending under three ion distribution conditions and without injection. f) Comparison of the change in radiative recombination rate under three ion distribution conditions and without injection.

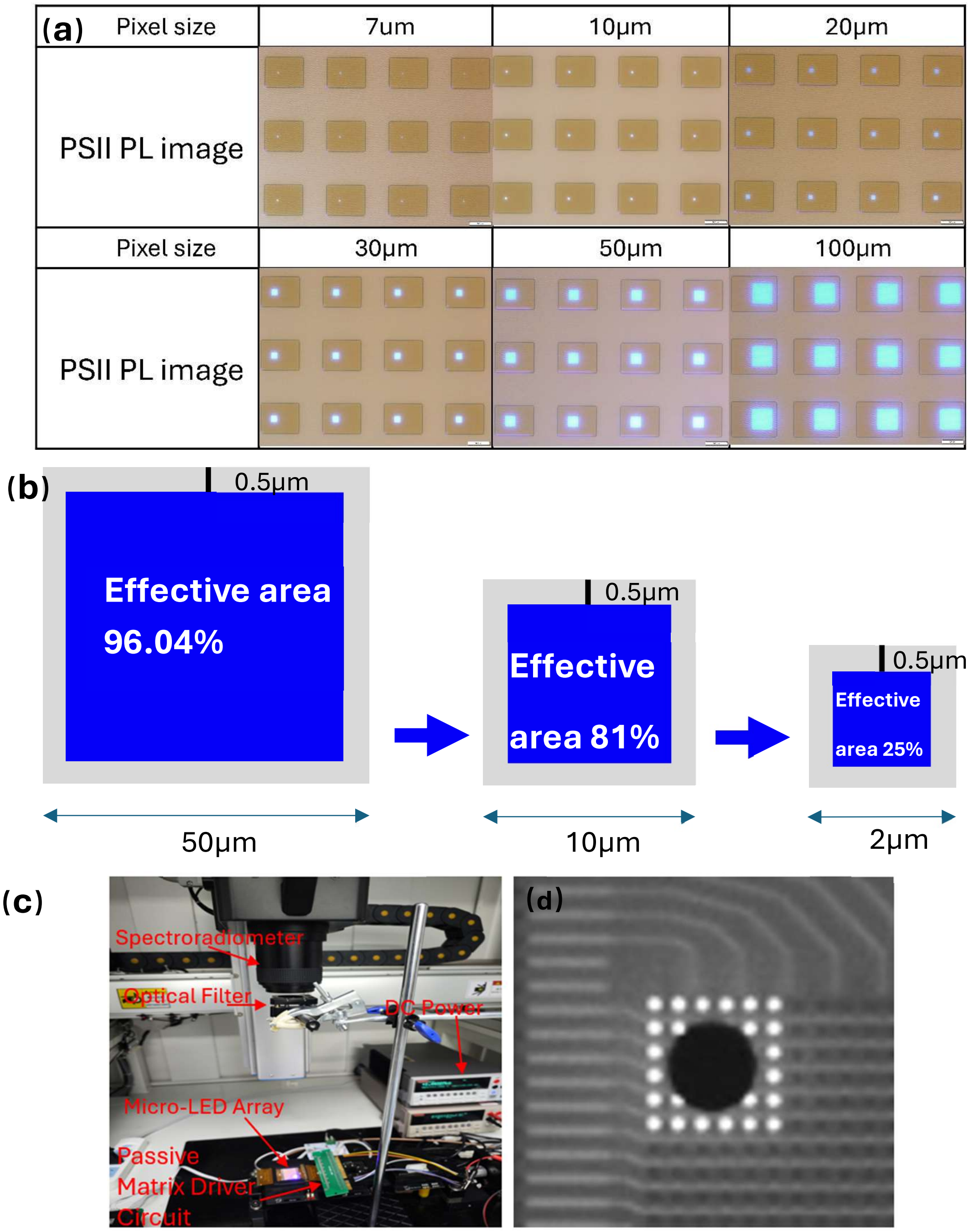

Figure S6. a) Photoluminescence micrographs of Micro-LEDs with different emission sizes. b) Traditional mesa etching causes plasma-induced damage to the pixel sidewalls, resulting in a reduction in the effective light-emitting area ratio. c-d) Test setup diagram and test area screenshot of the light-emitting performance of Micro-LED arrays.

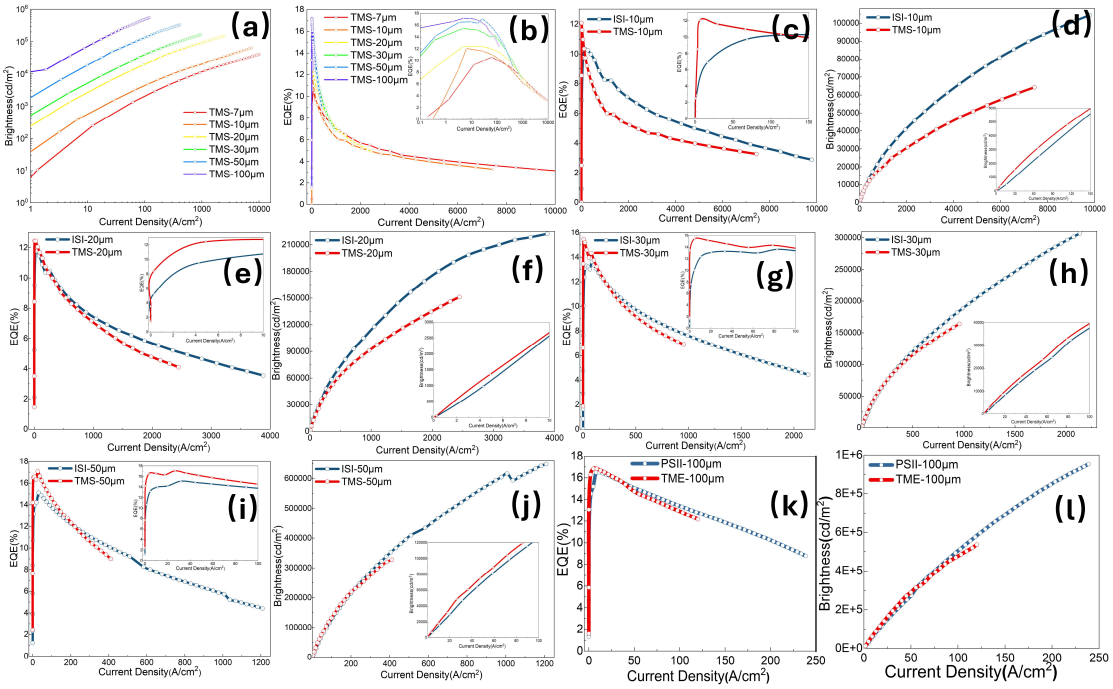

Figure S7. a-b) Luminance and EQE of Micro-LED chips with different emission sizes prepared by traditional mesa etching. c-l) Comparison of luminance and EQE of Micro-LEDs prepared by two isolation processes.

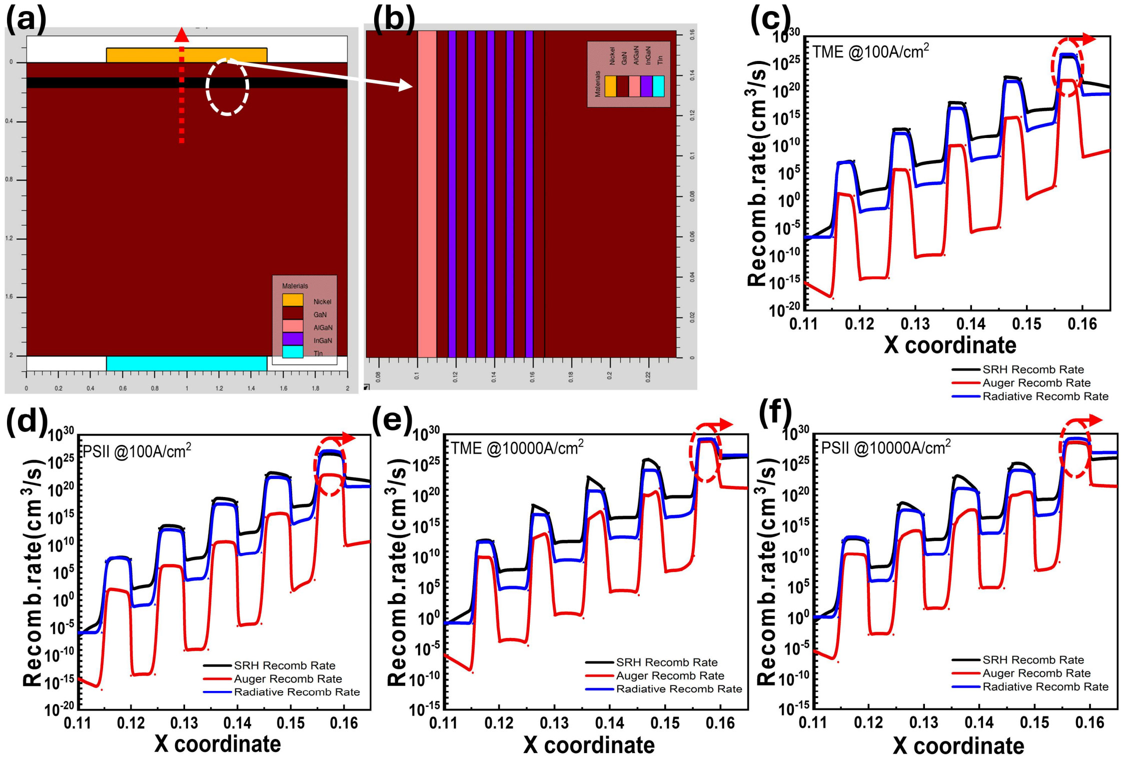

Figure S8. a-b) The schematic diagram of the LED structure used in the simulation, and the data was extracted from the multi-quantum well within the red circle. c-f) The study showed the recombination of charge carriers in the active region under 100A/cm^2 and 10000A/cm^2 current densities. Silvaco TCAD semiconductor simulation software was used for the modeling of Micro-LEDs with a 1 μm emission size for both TME and PSII strategies. The structure comprised a p-type layer (100 nm GaN + 10 nm AlGaN), an active region with a 5-period GaN (6 nm)/InGaN (4 nm) multi-quantum well (MQW), and an 1834 nm n-GaN layer. Carrier recombination in the active region was analyzed at current densities from 100 A/cm² to 10,000 A/cm², with data extracted from the MQW within the red circle.

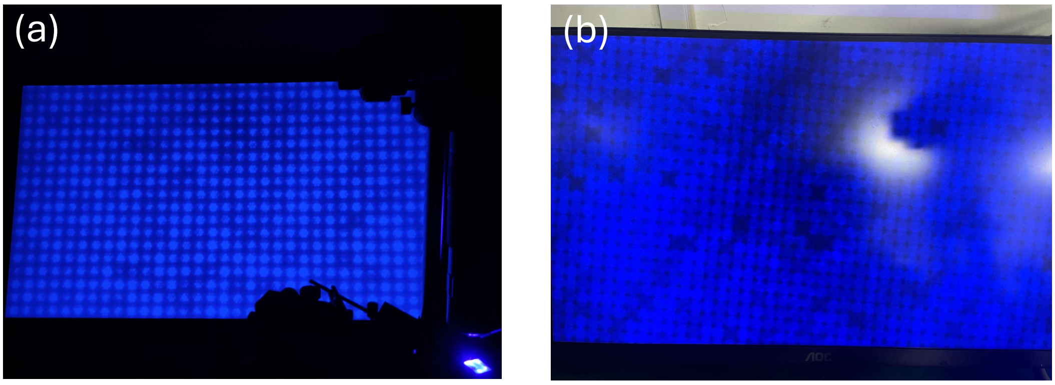

PSII-LED TME-LED

Figure S9. a-b) Compared with TME, the high-resolution active matrix-driven prototype based on the PSII strategy has a better suppression effect on light crosstalk and an improved contrast ratio.
